# A Bibliometric and Visual Analysis of Global Community Resilience Research

**DOI:** 10.3390/ijerph182010857

**Published:** 2021-10-15

**Authors:** Qiaoyun Yang, Dan Yang, Peng Li, Shilu Liang, Zhenghu Zhang

**Affiliations:** 1 Faculty of Humanities and Social Sciences, Dalian University of Technology, Dalian 116024, China; qiaoyunyang@dlut.edu.cn (Q.Y.); 18847130748@163.com (D.Y.); liangshilu@mail.dlut.edu.cn (S.L.); 2 School of Civil Engineering, Dalian University of Technology, Dalian 116024, China

**Keywords:** community resilience, urban development, bibliometric analysis, hotspots

## Abstract

Resilience is an important issue in urban development, and community resilience (CR) is the most typical representative in building urban resilience, which has become the forefront of international resilience research. This paper presents a bibliometric and visual analysis of community resilience research collected from the WoS Core Collection database over the past two decades. H-index, citation frequency, centrality and starting year were adopted to analyze the research objects by bibliometric tools including CiteSpace, VOSviewer, and Gephi. The national and institutional characteristics of macro-geographical distribution and the characteristics of disciplines, journals, authors, and author cooperation of micro-knowledge network distribution were revealed. Finally, the potential research directions of community resilience in the future were discussed. The results show that there are three stages in community resilience research. Seven intellectual bases constitute the research background for community resilience, including social capital mechanism, the evolution of resilience knowledge, earthquake resistance and disaster mitigation, substance abuse, resilient development in rural communities, resilience-building in the least-developed countries, and emergency preparedness. Our analysis shows that the hottest community resilience research topics are the concept of resilience, climate resilience, the social capital mechanism, macro-environment and disaster-reduction policies, and an evaluation index system for community resilience.

## 1. Introduction

The concept of resilience originated from the Latin word “resilio”, which means “bounce back”. The term resilience is commonly used to describe the ability of an entity or system to return to a normal state after the occurrence of an event that disrupts its state [1,2]. In the 1970s, it became an important area of research in ecosystems, and then the concept of resilience gradually expanded from ecosystems to social systems. From the perspective of resilience development, its connotation has transformed from engineering resilience to ecological resilience to evolutionary resilience [3]. Engineering resilience believes that there is one and only one stable state, focusing on the ability of the system to return to its original state [3]. Ecological resilience considers that some systems have multiple stable states, so the magnitude of the disturbance they can withstand before changing their structure is used to measure resilience [4]. Evolutionary resilience has received extensive attention from the academic community in recent years and emphasizes the ability of the system to change, adapt, and change in the whole process of responding to pressures and constraints [5]. Recently, with the development of human practice in response to disasters, policies and measures based on the concept of resilience have been put forward successively around the world to mitigate disaster risks. Resilience-building has also become the main disaster mitigation idea in many countries. In the UN 2030 Agenda, 6 out of 17 sustainable development plans mentioned the proposition of resilience [6].

Community is a basic unit of human group activities. Building community resilience is the basis for realizing urban resilience and regional resilience. In the past two decades, community resilience has become a new direction of community development [7]. Under the superposition of multi-disaster risk factors, the importance of community self-organization and management is further highlighted. Particularly, in the context of the COVID-19 epidemic, the occurrence of geo-hazards such as a tropical storm, earthquake, or wildfire will dramatically complicate emergency response efforts and the management of evacuees and medical resources. Several natural disasters have already occurred during the COVID-19 crisis, and as the crisis is expected to persist, various seasonal natural disasters are expected to inflict concurrent multi-hazard events around the world [8]. We need to plan for and be able to respond and adapt our response during a multi-hazard crisis. A central component of risk reduction is building communal resilience—the ability of a community to work together to prepare for, cope with and recover from a disaster [3,9,10]. Previous studies on community resilience mostly focused on identifying and analyzing the concept of community resilience [11,12,13], community resilience assessment models, and index measurement [14,15,16,17]. There are few comprehensive bibliometric studies on the literature on community resilience. 

The objective of this study is to present a bibliometric and visual analysis of the past two decades of community resilience research. This paper explores the following five questions: (1) What is the overall publication trend of community resilience research in the world? (2) Which countries or regions have been dominant in the field of community resilience? (3) Which disciplines, journals, institutions, and authors in community resilience research are the most influential? (4) What are the most important intellectual bases and research hotspots in community resilience research? (5) What is the future development trend of community resilience, and what suggestions can be made to scholars and policymakers?

## 2. Methodology and Data Acquisition

### 2.1. Methodology

Bibliometrics methods and knowledge mapping visualization software were utilized to present the knowledge distribution and emerging trends of community resilience research from different aspects. Bibliometrics was first introduced by Pritchard [18], which can reveal the research characteristics of a specific field through quantitative and statistical methods. The bibliometric method has been applied in many fields, e.g., construction safety management [19], building information modeling (BIM) [20], open innovation [21] and tourism [22]. So far, it has become an important research tool in various fields. Scientific mapping is an important step in bibliometric analysis [23], which can objectively present the research status of a discipline [24]. There are many visual software to assist bibliometric analysis. Visualization maps in this study were mostly generated by CiteSpace (Chaomei Chen, Philadelphia, PA, USA) [25,26,27] and VOSviewer (The Centre for Science and Technology Studies, CWTS, Leiden, The Netherlands) [28,29,30] software. In addition to providing information on major thematic focus areas, these tools can also be used to untangle complex interrelationships between different underlying components of a research field [31]. CiteSpace is an efficient information visualization tool, which was developed by Professor Chen Chaomei of Drexel University (Philadelphia, PA, USA). It is a free JAVA application that is used to visualize and analyze trends and patterns in the scientific literature [32]. In this paper, CiteSpace was adopted to generate knowledge maps of country, category, document co-citation cluster, and high-frequency keywords cluster. VOSviewer, developed by Eck and Waltman (The Centre for Science and Technology Studies, CWTS, Leiden, The Netherlands), is a visualization software with powerful functions and a user-friendly interface in co-occurrence analysis and co-citation analysis [33]. VOSviewer was used to implement co-citation journal, author cooperation, and author co-citation network in this study. Additionally, ArcGIS (Esri, RedLands, CA, USA) and Gephi (The Gephi Consortium) were used to perform the global distribution of publications and the cooperation network between research institutions.

**Figure 1:**
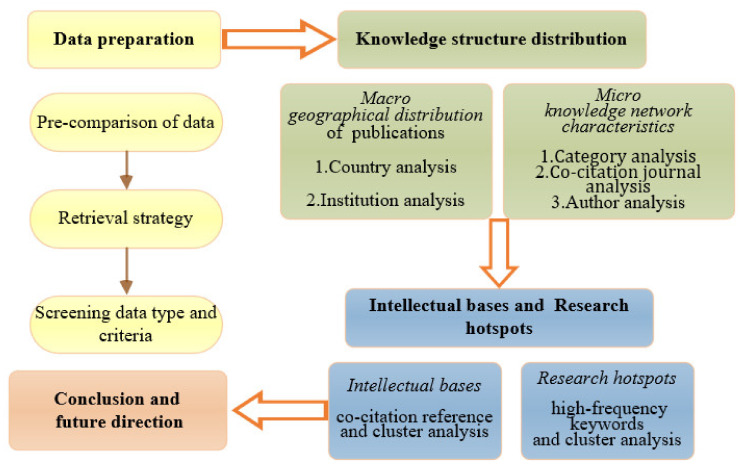
Bibliometric and visualization analysis framework of community resilience research.

The study framework of this paper is shown in Figure 1. First, to obtain comprehensive and representative data, the search terms were pre-analyzed during the data preparation stage. After the retrieval strategy was determined, the data were screened according to publication types and criteria. Based on the visualized network and frequency statistics, the macro-geographical distribution of community resilience research was investigated in terms of countries and institutions. The micro-knowledge distribution characteristics were analyzed by disciplines, journals, and authors. Subsequently, intellectual bases and research hotspots were revealed through the co-occurrence and cluster analysis of references and keywords. Finally, the potential research directions in community resilience were discussed.

### 2.2. Data Acquisition

For most subjects, the Web of Science (WoS) is the most authoritative data source used to study publications because it contains the most important and influential journals throughout the world [34,35]. Therefore, articles related to community resilience from the WoS Core Collection were selected as the database of this study. Detailed information of literature, e.g., author, institution, publication year, and journal source, were extracted from the WoS. The retrieval condition was designed as follows: TS (Topic) = (“community resilience*” OR “resilient commuit*”) AND document types = article AND language = English AND Timespan = 2001–2020. The download date was 22 April 2021. In total, 2266 valid literature in the community resilience research were acquired.

## 3. Distribution Characteristics of Community Resilience Research

### 3.1. Temporal Distribution of Community Resilience Research

**Figure 2:**
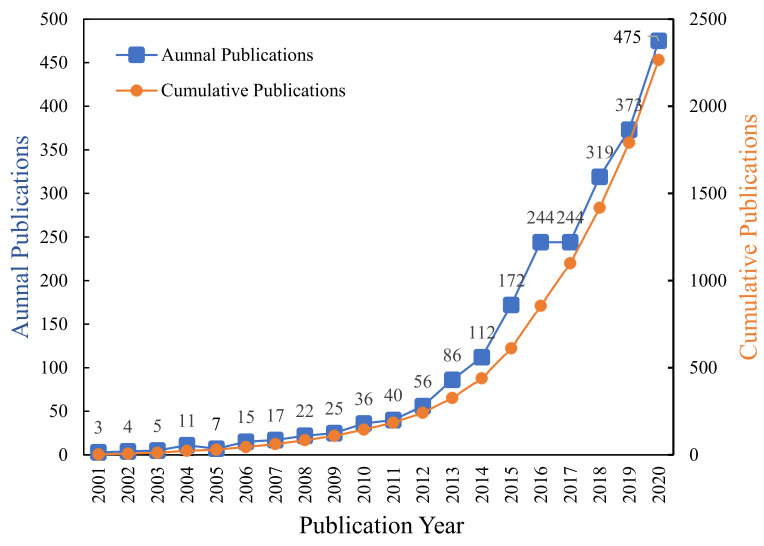
Annual publication distribution in community resilience research over the past two decades.

Among the 2266 literature, the earliest publication time was April 2001, while the latest publication time was December 2020. The quantity of the publications is an important indicator that reveals the development trends of scientific research [36]. The distribution of annual publications in the last 20 years is shown in Figure 2. The number of publications in community resilience research has shown a gradual increase in the past 20 years, indicating that community resilience research has attracted increasing attention around the world. The number of publications increased slowly from 2001 to 2009, and community resilience research was in its infancy with an average annual publication volume of only 14.5. In 2010, UNISDR launched the “Making Cities More Resilient” campaign. Since then, the number of publications has increased slightly from the original basis. In 2013, the Rockefeller Foundation launched the world’s first “Global 100 Resilient Cities” project. Under its impetus, there was significant growth in the number of publications from 2013 to 2020. During this period, publication output accounted for 89.36% of the total. In 2020, 475 articles were issued, an increase of 158.33 times over 2001. 

The past 10 years are the main period for global community resilience research. To be specific, the period from 2010 to 2012 is the slow growth stage, whereas the period from 2013 to 2020 is the rapid growth stage. The entire publications reviewed, 2266 papers, had received a total of 41,323 citations with an average of 18.24 citations per paper. In the same period, 7556 articles on ecological resilience were retrieved, with an average citation frequency of 32.1. There were 2252 articles on engineering resilience retrieved with an average citation frequency of 19.8. It was found that the average citation frequency in community resilience was lower than that of ecological resilience and engineering resilience over the past two decades.

### 3.2. Macro Global Geographic Distribution of Community Resilience Research

#### 3.2.1. Distribution of Countries/Regions

**Figure 3:**
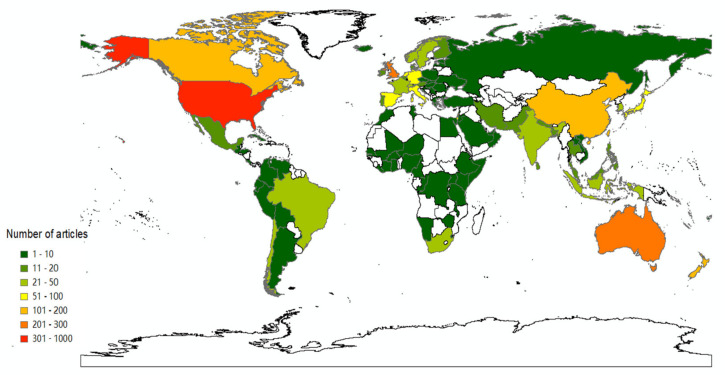
Geographical distribution of publications in the community resilience research from 2001 to 2020. The map was created by ArcGIS 10.2 software. Red areas indicate the most publications and dark green areas indicate the fewest publications. Blank areas mean that no relevant literature data has been collected.

There are 115 countries or regions that have engaged in community resilience research over the past twenty years. The data was exported by CiteSpace software and then a global geographic distribution heat map was drawn by ArcGIS 10.2 software, as shown in Figure 3. The United States and Canada in North America, the United Kingdom in Europe, China in Asia, and Australia in Oceania have published more than 100 articles in community resilience research. These areas are called hot spots for the community resilience field. It can be seen from Figure 3 that many African countries and regions have a blank state of research on community resilience, such as Algeria, Libya, and Sudan. South Africa is the most concerned about community resilience among African countries, which has published about 35 related articles since 2004. Other African countries began conducting research on community resilience around 2010, while several countries represented by Somalia, Dem Rep Congo, and Tunisia have only paid attention to community resilience building recently. From 2004 to 2005, countries such as Bolivia, Brazil, and Chile in South America carried out earlier studies on community resilience, while Venezuela and Ecuador only started studies on community resilience after 2015. Guyana, Uruguay, and Paraguay, located in South America, have no relevant research on community resilience in the collected databases. Most South American and African countries still need to further strengthen their studies, so that community resilience research can receive more attention on a global scale. 

**Figure 4:**
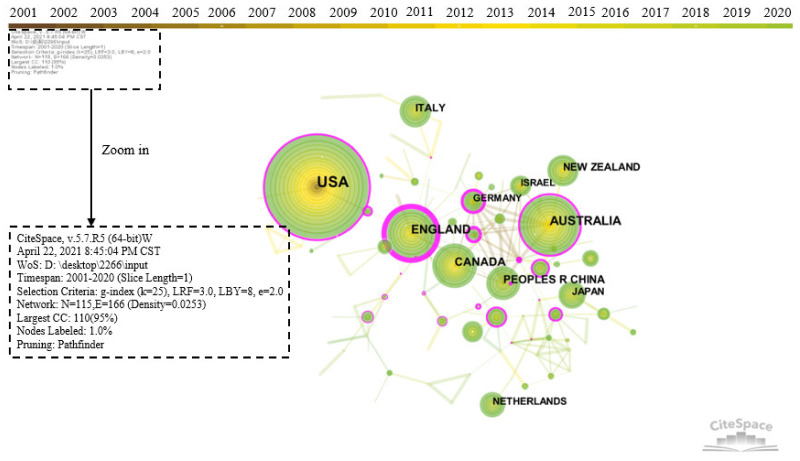
Visualization map of countries/regions cooperation network in the community resilience research from 2001 to 2020. Note that the size of the circle refers to the number of publications. The lines between the circles indicate cooperative relationship and the thickness indicates the strength of links between the countries/regions. The thickness of the outer pink circle represents the size of centrality.

The country node in the CiteSpace software was selected to generate the national or regional cooperation network map, as shown in Figure 4. Years per slice were set to 1. The larger the circle, the more the number of publications. The lines between the circles indicate cooperative relationship and the thickness indicates the strength of links between the countries or regions. Network density refers to the ratio of the actual number of lines to the theoretically maximum number of lines in a network. Betweenness centrality is an index to measure the importance of nodes in a network. CiteSpace uses this indicator to discover and measure the importance of documents and uses pink circles to highlight such documents (or authors, journals, institutions, etc.) [37]. The thicker the outermost circle, the larger the centrality, which means that cooperation between the countries or regions is frequent. According to Figure 4, the cooperation between countries or regions is generally close, with a network density of 0.0253.

The top 10 most productive countries or regions are listed in Table 1. The United States ranks first in the number of publications with 875, accounting for 28.65% of the total. This is followed by Australia, with 287 papers, accounting for 9.40% of the total outputs. England occupies third place, with 207 publications, accounting for 6.78% of the total number of outputs. Countries or regions that have published more than 100 articles also include Canada, China, and New Zealand. According to centrality, England has the thickest outer circle in Figure 4, with a centrality of 0.94, indicating that it plays the most important role in the knowledge transfer process of community resilience research. In addition to England, countries with a centrality greater than 0.25 contain Germany, Australia, and the USA. The average citation frequency of articles published in the United States is 24.19, ranking first, indicating that the United States has high authority in community resilience research and has been widely recognized by academia. The average citation frequencies of England, Canada, and New Zealand are also at the forefront and these countries or regions have an important influence on community resilience research. In terms of the year when the study began, the United States and New Zealand were the earliest, in 2001. There is an overall positive correlation between the starting year and the average of citations per paper (TC/P) for a country. This is because the earlier the publication, the more likely it is to be cited. Overall, many countries or regions tend to cooperate and communicate with each other. The community resilience research has strong global network characteristics.

#### 3.2.2. Distribution of Institutions

**Figure 5:**
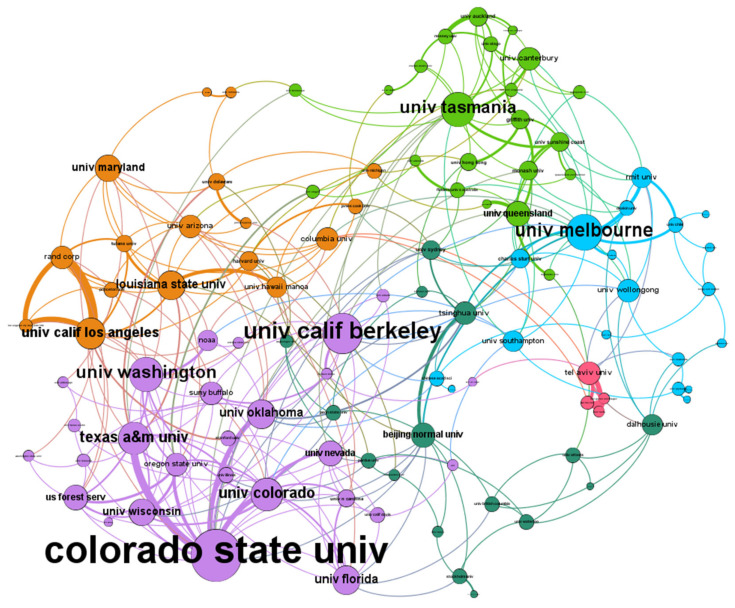
Visualization map of cooperation network between high-yield institutions in community resilience research from 2001 to 2020. Note that the size of the circle represents the weight of the connection and the thickness of the connection represents the number of cooperation.

To present the cooperation overview clearly, Gephi software was used to present the cooperation network map of high-yield institutions with more than eight articles, as shown in Figure 5. The size of the circle represents the weight of the connection and the thickness of the connection represents the number of cooperations. Colorado State University, the University of California Berkeley, and the University of Melbourne have the most extensive cooperation. Overall, the characteristics of cooperation among high-yield institutions are remarkable.

The top 10 productive institutions in community resilience research are listed in Table 2. Colorado State University is the most productive with the most extensive cooperation, with 51 articles published. Texas A&M University is the second-ranked institution with 38 articles published, followed by the University of Queensland and Louisiana State University. It is worth mentioning that the average citation frequency of the University of Washington, Louisiana State University, and the University of Queensland are as high as 67.56, 32.86, and 30.65, respectively. It can be concluded that the quality of contributions of the three institutions is high, which can be widely referenced by scholars in this field. The top 10 institutions also include Oregon State University (Corvallis, OR, USA), the University of Melbourne (Melbourne, Australia), University of California Los Angeles (Los Angeles, CA, USA), RAND Corp (Santa Monica, CA, USA), and Oklahoma State University (Stillwater, OK, USA).

### 3.3. Distribution Characteristics of the Micro-Knowledge Network of Community Resilience Research

#### 3.3.1. Distribution of Categories

**Figure 6:**
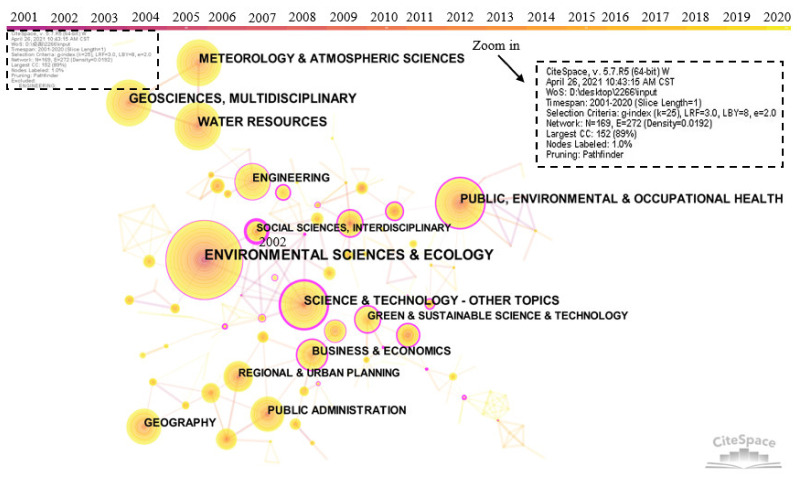
Network visualization map of categories in community resilience research from 2001 to 2020. Note that the size of circle refers to the number of articles. The thickness of the outer pink circle represents the size of centrality.

As shown in Figure 6, the co-occurrence mapping of the category network was generated by CiteSpace to present a clearer interdisciplinary situation. Years per slice were set to 1. Table 3 lists statistical indicators of the top 10 categories. It is found that community resilience research is a multi-disciplinary research field, among which the number of articles published in environmental science and ecology ranks first with 691 articles. The second discipline is water resources with 328 articles published. The third-ranked discipline is Earth Science and Multidisciplinary Research, with 302 articles published. The top 10 disciplines also include Meteorology and Atmospheric Sciences, Science and Technology, Public, Environmental and Occupational Health, Engineering, Geography, Public Administration, and Business and Economics.

Science and Technology—Other topics rank first in centrality with 0.45. This is followed by Public, Environmental and Occupational health with a centrality of 0.32. The disciplines whose centralities are more than 0.15 contain Business and Economics, Engineering, and Environmental Sciences and Ecology. Judging from the year when the research was started, Business and Economics was the latest, while other subjects started very early. In terms of the average citation frequency of disciplines, Geography, Public Administration, and Engineering rank as the top 3 with more than 20 citations, indicating that these three disciplines have contributed a high-quality knowledge base to community resilience research in the past 20 years. They have gradually become more active categories in community resilience research.

#### 3.3.2. Distribution of Co-Citation Journals

**Figure 7:**
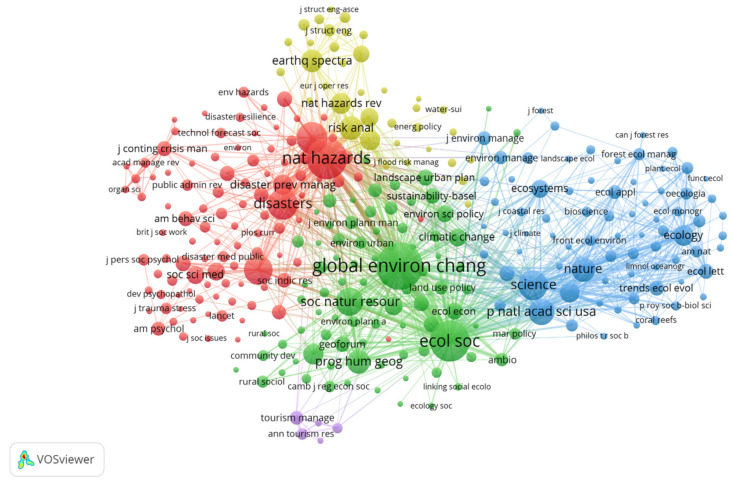
Network visualization map of co-citation journals in community research from 2001 to 2020. Note that the lines between the circles represents co-citation relationship. The thickness and number of connections between the nodes indicate the strength of links between journals.

Journal co-citation occurs when two pieces of literature published in different journals receive a citation from a third literature of another journal [38]. Co-citation analysis of journals was performed using VOSviewer. When the minimum number of citations of a source was set to 50, 304 nodes were generated. The cluster resolution was set to one and the minimum cluster size was set to two. The network visualization map of co-citation journals in community research from 2001 to 2020 is shown in Figure 7. Clusters, which are symbolized by different colors, were generated based on citation links. The size of the circles indicates the relevance of topics [39]. The line between the circles represents a co-citation relationship. The thickness and number of connections between the nodes indicate the strength of links between journals. Table 4 displays the top 10 highly cited journals and their statistical parameters in the community resilience research. Global Environmental Change—Human and Policy Dimensions hosted by England is the most influential journal in terms of citation frequency. This journal has a total citation frequency of 2257 times and total link strength of 82,562. Both indexes are much higher than other journals, indicating that the journal has the highest recognition and authority in community resilience research. It is followed by Natural Hazards, Ecology and Society, International Journal of Disaster Risk Reduction and Disasters. All the journals have more than 1000 citations, and the total connection strengths are more than 30,000, indicating that the above journals have a high contribution and influence in community resilience research.

Among the top 10 high-cited journals, five are from the USA, three from the UK, one from the Netherlands, and one from Canada, revealing that North America, represented by the United States and Canada, and Europe, represented by the United Kingdom and the Netherlands, are the main concentrated areas of community resilience research. They have a strong research foundation in the community resilience field.

#### 3.3.3. Distribution of Author Collaboration

**Figure 8:**
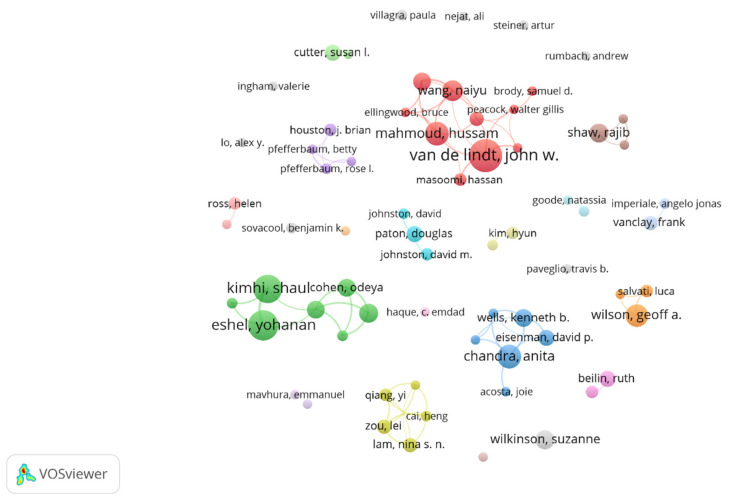
Visualization map of main author cooperation network in community resilience research from 2001 to 2020. The size of the circle refers to the number of articles published by the author.

The number of authors with more than five articles was counted, and a visualization map of the main authors’ cooperation network was drawn by VOSviewer software, as shown in Figure 8. The cluster resolution was set to one and the minimum cluster size was set to two. The minimum number of citations of an author was set to 0. Table 5 summarizes the top 10 productive authors with statistical characteristics, including publications, institution, country, and H-index. John W.van de Lindt is the author with the largest quantity of publications, with a total of 20 articles, and the initial publication year was 2016. Shaul Kimhi, Yohanan Eshel, and Hussam Mahmoud tied for second place with 14 papers. Other authors who have published more than 10 articles are Naiyu Wang, Anita Chandra, and Peihui Lin. It can be seen from the initial publication years of the high-yield authors in Table 5 that the top 10 productive authors in the field of community resilience became active after 2010 and achieved prominent achievements. This further indicates that the recent decade is a critical period of community resilience research and the last five years are more significant.

According to Figure 8, there are multiple potential cooperation teams in the author’s cooperation network. Especially, there are three noteworthy cooperation teams in the author’s cooperation network, which are represented by red, green, and blue networks. The first team is the research team (red) represented by John W. van de Lindt and Hussam Mahmoud. The second is the research team (green) represented by Shaul Kimhi and Yohanan Eshel. The third is the research team (blue) represented by Anita Chandra and Kenneth B. Wells. The collaboration among the three teams was extensive and productive. However, the authors’ cooperative network is relatively loose overall. Therefore, cross-institutional and cross-border collaboration between high-yield authors in community resilience research needs to be further strengthened.

From the perspective of the distribution of high-yield authors, Colorado State University in the United States, Tel Hai Academy College in Israel, and Zhejiang University in China are well-known universities in the community resilience field that are rich in high-yield authors and have a strong research foundation in community resilience research.

**Figure 9:**
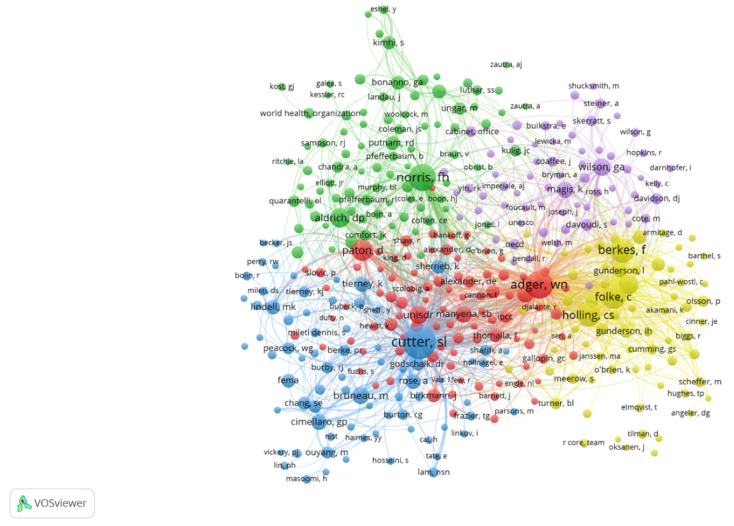
Network visualization map of co-cited authors in community resilience field from 2001 to 2020. Note that the size of the node represents the author’s citation frequency and the thickness of lines between two nodes refers to the co-citation frequency.

Authors whose citation frequency is more than 25 were selected by the VOSviewer software and then the co-citation network of authors is shown in Figure 9. The cluster resolution was set to one and the minimum cluster size was set to two. The larger the node, the higher the author’s citation frequency. The thicker the line, the higher the co-citation of the two authors. The publications of co-cited authors were roughly divided into five themes, symbolized by green, blue, red, yellow, and purple. Since community resilience is an interdisciplinary study, most related links between co-cited authors are cross-topic citations. It is found that Cutter S.L., Norris F.H., Adger W.N., and Folke C. have the most co-citation frequency among each other.

Table 6 presents the top 10 highly cited authors with characteristic parameters, including frequency, centrality, institution, starting year, and H-index. Among them, the most influential author in the community resilience field is Cutter S.L. from the University of South Carolina System, whose publications have been cited 658 times. This author’s earliest publication year in the community resilience field was 2008. His most influential article is “A place-based model for understanding community resilience to natural disasters” published in 2008. The author proposed a local disaster resilience model to improve the comparative assessment of disaster resilience at the local and community levels. Meanwhile, he also selected candidate variables for the inherent resilience measurement of the DROP model [14]. The citations of Norris F.H. from Dartmouth College were 640 times. The article “Community Resilience as a Metaphor, Theory, Set of Capacities, and Strategy for Disaster Readiness”, published in 2008 by Norris F.H. et al., provided a comprehensive theoretical perspective for understanding community resilience [40]. This article proposed that community resilience mainly comes from four aspects of adaptability, including economic development, social capital, information and communication, and community competence [40]. Adger W.N. from the University of Exeter has been cited 507 times, ranking third in the list of cited authors. His centrality is 0.25, which indicates that he plays a significant role in the knowledge connection in the community resilience field. The conference paper “Social Capital, Collective Action, and Adaptation to Climate Change” published by Adger in 2003 emphasized the considerable impact of collective action and social capital in the community’s response to climate change in the form of a case [41]. In addition to the authors mentioned above, the top 10 highly cited authors include Folke C., Berkes F., Holling C.S., Paton D., Walker B., Bruneau M., and Aldrich D.P.

## 4. Intellectual Bases and Hotspots of Community Resilience

### 4.1. Intellectual Bases of Community Resilience

**Figure 10:**
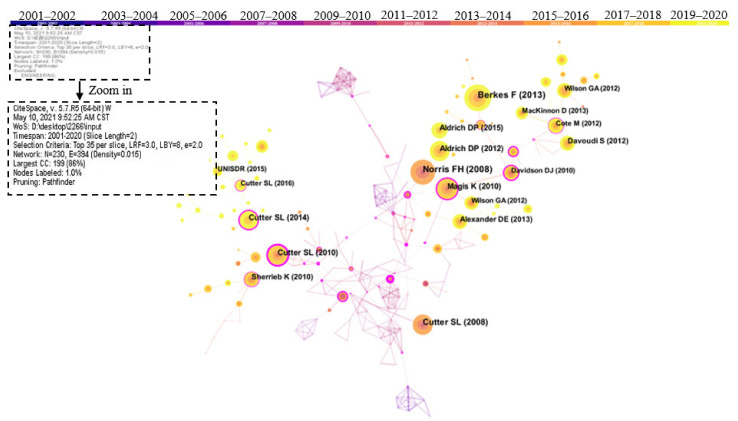
Network visualization map of co-citation documents in community resilience research from 2001 to 2020. Note that nodes refer to citations in data sources, and links represent co-citations between different documents.

The document co-citation network is shown in Figure 10 using CiteSpace software. The node type was set as a reference in this part. The time slice was set to 2 years, and the top 35 high-cited publications were selected for visual network presentation in each period. Nodes refer to citations in data sources, and links represent co-citations between different documents. The color of the link is consistent with the year color in Figure 10. For example, the yellow link represents a publication that was jointly referenced in 2019 and 2020. The top 10 highly cited documents in community research, acquired from the statistical results of references cited in 2266 articles, are listed in Table 7. The review “Community Security as a Metaphor, Theory, Set of Capacities, and Strategy for Disaster Readiness” was cited 244 times, the highest-frequency citation in the community resilience field. This paper believes that resilience and health stem from various adaptive abilities and define them as resources with dynamic attributes. The author combined this view with evidence to provide a better understanding and building community resilience [40]. The article called “Community Resilience: Towards an Integrated Approach”, published in Society and Natural Resources in 2013, was cited 217 times. This article explored comprehensive methods for constructing community resilience from the background of social-ecological systems, the psychology of development, and mental health. By integrating resilience research across disciplines, it emphasized the use of critical thinking in the construction of comprehensive resilience [42]. As the third most cited literature, the article “A place-based model for understanding community resilience to natural disasters”, published in 2008, has been cited 164 times. Overall, most of the highly cited documents are summary and commentary papers with a high reference value. 

The book “Building Resilience: Social capital in post-disaster recovery”, published in 2012, is the most-read book in community resilience research. Aldrich, the author of this book, examined the post-disaster responses of four distinct communities—Tokyo following the 1923 earthquake, Kobe after the 1995 earthquake, Tamil Nadu after the 2004 Indian Ocean Tsunami, and New Orleans post-Katrina. The book highlighted the critical role of social capital in the ability of a community to withstand disaster and rebuild both the infrastructure and the ties that are at the foundation of any community [43]. The article “Disaster Resilience Indicators for Benchmarking Baseline Conditions” has the highest centrality, indicating that it plays a crucial role in community resilience research. This article and the article named “A Place-based Model for Understanding Community Resilience to Natural Disasters” were published by the same author: Cutter S.L. Cutter et al. constructed a series of baseline indicators including social resilience, economic resilience, infrastructure resilience, and community resilience to show the spatial resilience distribution at the county level in the southeastern United States [15]. Additionally, these indicators were extended to three cities to present the comprehensive resilience score at the city level. This paper put forward some specific indicators for resilience assessment and provided a specific method for disaster planners and decision-makers to improve community resilience [15].

**Figure 11:**
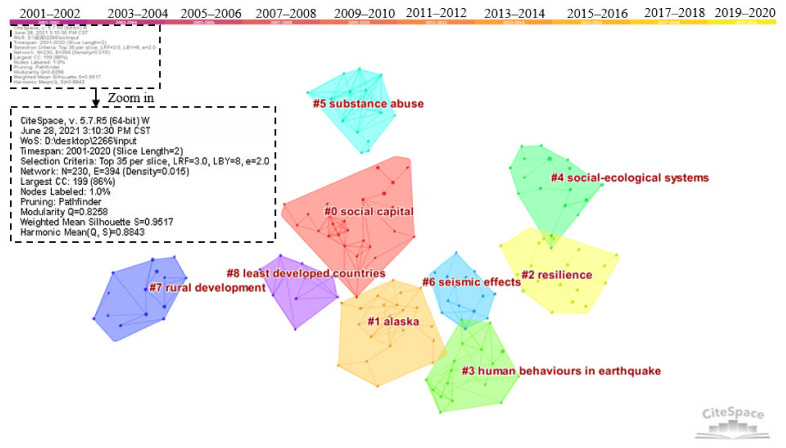
Cluster of co-citation document network.

Cluster analysis of highly cited documents was made by CiteSpace software, as shown in Figure 11. The time slice was set to 2 years, and the top 35 most cited documents were selected for each slice. As the founder and promoter of CiteSpace, Chen Chaomei suggested that 7–10 clusters are more suitable for overall structure distribution and content analysis, with 10 or more members in each cluster [44]. This is because we will not obtain a big picture if there are many clusters, and we will not also learn much information from the network if there are very few clusters [45]. Based on the network structure and the clarity of clustering, CiteSpace proposes two indicators: the modularity value and the silhouette value, which can be used as the basis for us to judge the effect of spectrogram drawing [44]. The ranges of silhouette and modularity values are from 0 to 1. The larger the silhouette, the more perfect the clustering. The silhouette of each cluster should be above 0.7 [46]. The clustering in this study is based on the goodness of fit between the research content and 9 clusters composed of 10 or more components were obtained (as shown in Figure 11). The mean silhouette of clusters is 0.8843, and all the silhouette values of each part are above 0.7. Modularity is a crucial parameter to measure the structural characteristics of the overall clustering network [46]. The modularity of the clustering is 0.8258, and the fitting effect is preferable. After clustering content integration, intellectual bases of community resilience were finally classified into seven classes. It should be noted that Alaska (#1) is a regional amalgamation of studies, and it is not viewed as one of the classes. This is because the studies related to Alaska are included in the following classes.

Class one: Social capital mechanism (#0). This class has the largest area with a silhouette of 0.979 and contains 27 papers. Compared with the previous emphasis on physical resilience, scholars have gradually realized the key role of social capital and its network in disaster recovery. The social capital network of individuals and communities is an important way to obtain various resources in a disaster situation, including information, aid, financial resources, and emotional and psychological support. [47,48,49]. Most of the early studies focused on specific disaster cases and explored the impact of social capital and the presence of networks to call on authorities to consider social capital as a significant aspect of community resilience. In recent years, the role of social capital mechanisms in community resilience-building has become more prominent, and the research has become more in-depth.

Class two: Evolution of Resilience Knowledge (#2 and #4). The silhouettes are 0.986 and 0.914, and the quantities of articles are 21 and 18, respectively. The transfer of resilience knowledge in different disciplines has become a significant knowledge background for current resilience research. In 1973, Holling officially introduced the concept of resilience into the ecosystem for the first time, regarding it as the ability to persist in the face of change [3]. Subsequently, resilience extended to psychiatry and psychology [50,51], laying a good foundation for the research of psychological resilience. After the twentieth century, resilience has gradually been adopted in the fields of ecology, psychology, economics, and engineering. As the idea of resilience gradually attempts to become integrated on the social level, some scholars have proposed that there is a large degree of coupling between social systems and ecosystem resilience construction. Additionally, there are many obvious connections between them, especially those groups and communities that rely on ecology and environmental resources for their livelihoods [52]. Folke described the emergence of a dynamic perspective of social ecosystems in the construction of resilience. He also proposed establishing an adaptive management method that responds to changes in the ecosystem [53]. The emergence of the social ecosystem perspective provided a vital resilience analysis framework for later academic circles and enriched the theoretical and practical achievements of resilience research.

Class three: Earthquake resistance and disaster mitigation (#3 and #6). The silhouettes are 0.893 and 0.952, and the number of articles is 19 and 15, respectively. These studies are mainly concentrated in the field of specific disaster risk resilience, especially resilience focusing on specific natural disasters such as earthquakes, volcanic eruptions, floods, tsunamis, and hurricanes. These studies target specific areas for resilience assessment, or target resilience measurement and improvement of a single subsystem, such as electric power systems [54], medical infrastructure, and economic systems [55,56]. The expansion of resilience-building based on specific disaster areas provides a reference for the follow-up practice of communities to resist specific risks.

Class four: Substance abuse group research (#5). The silhouette is 0.982 and contains 15 articles. The early studies on this topic mostly focused on substance abuse by special groups such as African Americans, children, and adolescents [57,58]. To improve the resilience of special groups to resist substance abuse, researchers generally use interview-style methods for special groups to discuss measures to build resilience from the perspective of families, communities, schools, and clinics. However, with the changes of the times, the topic has gradually expanded to new groups that have attracted broad attention, such as transgender populations and veterans [59,60].

Class five: Rural community resilience development (#7). Its silhouette is 0.987 and it contains 14 papers. With the acceleration of global urbanization, the disappearing rural areas and their weak economy, and declining population make it more urgent to enhance rural resilience. This type of paper focuses on the resilience of rural communities and remote rural areas that respond to specific disaster risks. Through field surveys of people in rural areas, scholars explored the factors that influence the resilience of rural communities, including community members, social, economic, and environmental factors [61,62], and put forward suggestions to policymakers to enhance the community’s ability to withstand disasters. This type of study mostly uses empirical methods such as questionnaires and interviews. The research results have strong explanatory power.

Class six: Resilience-building in the least-developed countries (#8). The silhouette of this cluster is 0.968 with 10 articles. The least developed countries have weak anti-risk capabilities in certain areas such as society, economy, politics, and the environment, which have attracted widespread attention from academic circles and international organizations. Countries and regions with weaker climate change resilience are the focus of the international community. International organizations usually establish special funds to enhance regional climate resilience, such as the GLOF Risk Reduction Project of the United Nations Development Program and the Reducing Climate Change-induced Risks and Vulnerabilities from Glacial Lake Outburst Floods in the Punakha-Wangdue and Chamkhar Valleys funded by the Global Environment Facility (Washington, DC, USA) [63]. Similar research also includes exploring resilient development paths in areas of armed conflict and economically underdeveloped areas [64,65]. This type of article mostly conducts case studies in underdeveloped countries and regions to provide different dimensions of resilience-building suggestions for regions with weaker risk resistance. 

Class seven: Emergency preparedness (#9). It contains 10 articles with a silhouette of 0.964. Adequate emergency preparedness is viewed as an essential element of disaster response and recovery. Preparing for disasters, such as emergency material storage and evacuation plans, can greatly reduce losses caused by the disaster; with the increasing uncertainty of natural and unnatural disasters, more and more scholars have focused their disaster preparedness on the level of families and special groups. More people realize that in addition to the measures of emergency management departments, the emergency preparedness of individuals and families is also crucial. Emergency preparedness is complex and requires sufficient knowledge, motivation, resources, and education to promote preparations. Furthermore, there is an urgent need to move forward in the direction of focusing on the unique needs of children, the elderly, and people with functional impairments [66,67]. This type of research believes that expanding emergency preparedness at the individual and family level to the community level can strengthen community resilience.

### 4.2. Research Hotspots of Community Resilience

**Figure 12:**
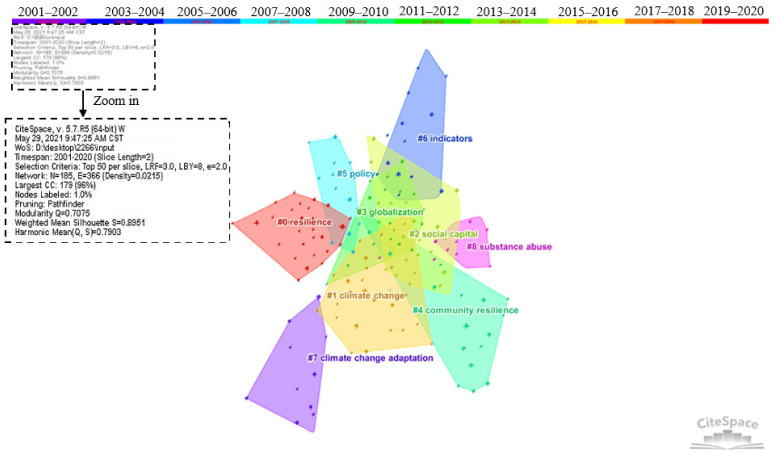
The cluster network mapping of high-frequency keywords.

The cluster network mapping of high-frequency keywords was conducted by CiteSpace. The time slice was set to 2 years, and the top 50 high-frequency keywords were extracted to form clusters. The high-frequency keywords were sorted into nine specific clusters, as shown in Figure 12. The average modularity and silhouette of clustering in Figure 12 are 0.7075 and 0.8951, respectively. The overall clustering effect is good. Due to the length limit, only the top 30 high-frequency keywords are presented in Table 8. It was found that excluding the keyword community resilience, the top five high-frequency keywords are vulnerability, climate change, disaster, framework, and adaptation. How to deal with the vulnerability of communities, adapt to climate change, improve the framework for building resilience, and resist various uncertain disaster risks have become the key issues in community resilience. The top 10 high-frequency keywords also include risk, management, recovery, model, and system. After the clustering of high-frequency keywords, knowledge divisions of community resilience research were carried out. Note that the topic of substance abuse (cluster eight) constitutes continuous stresses and requires ongoing community support of families and individuals, but this is not an integral part of the study of community resilience. Therefore, the topic of substance abuse is not viewed as a research hotspot. Finally, all clusters identified by CiteSpace were further summarized into the five hotspots, and detailed analyses were as follows:

Topic one: The concept of resilience (cluster zero and cluster four).

Resilience was first introduced to the field of ecosystems by the ecologist Holling C. and later expanded to sociology. It has experienced a process from engineering resilience to ecological resilience to evolutionary resilience and has become a hot topic of multidisciplinary joint research [3]. However, this has also led to different interpretations of resilience in different disciplines. Reaching a consensus on these concepts is still an urgent issue facing the academic community. Therefore, many scholars have turned their attention to the sorting out of resilience and related concepts. Folke C. has systematically integrated “resilience”, “adaptability”, and “transformability”. He argued that adaptability represents the capacity to adjust responses to changing external drivers and internal processes and is part of resilience. Transformability is the capacity to cross thresholds into new development trajectories. All three are critical factors influencing the transformation of the social–ecological systems (SES) [5]. Manyena SB re-examined the role of resilience and vulnerability on the easily confusing problem of the concept of resilience, and explained the relationship between vulnerability and resilience, making the concept of resilience clearer [11]. With the continuous extension of the concept of resilience, community resilience, as the foundation of urban resilience construction, has become the main direction of community development in many countries today. However, many scholars still misuse and confuse community resilience in community building. By combing and comparing the concepts of community resilience, community institutions, community vulnerability, community adaptability, and community capacity, David Matarrita-Cascante et al. explained the differences and connections among these concepts and further clarified the dominant role of community resilience in disaster response [12]. Evangelos Ntontis et al. examined how community resilience was used in the UK document on guiding floods to explore how different texts define community resilience [68]. This study highlighted community resilience’s procedural and dynamic properties and pointed out the main priorities for strengthening community resilience policies, which provides policy suggestions for policymakers [68]. The meaning and characteristics of resilience are increasingly clarified with the development of systematic community resilience.

Topic two: climate resilience (cluster one and cluster seven).

Climate change is a common challenge facing human society. As societies’ basic spatial-demographic units, communities play a key role in the response to climate change. Climate resilience has also become a vital aspect of community resilience research and practice. For many years, “mitigation” and “adaptation” have been considered as the main strategies to combat climate change [69]. In the study of dealing with community climate change, academia often takes case studies as a general research method to discuss a community’s climate response. Berkes F. and his collaborators conducted a study on the small community of Sachs Harbour in Canada’s western Arctic from the perspective of socio-ecological resilience. The short-term adaptation mechanisms and long-term adaptation strategies of local residents provide references for other regions to combat climate change. Newly developed co-management institutions can provide opportunities for feedback and connections between different levels, which can improve the learning and self-organization capabilities of the community [70]. Based on a survey of poor rural areas in South Africa, Anele Mthembu et al. analyzed the biophysical and socio-economic aspects of the region and proposed a bottom-up, proactive, and systematic approach to manage climate-vulnerable areas [71]. Based on a bibliometric analysis of the literature on mitigation, adaptation, and resilience related to climate change, Rachel Einecker et al. suggest that a high level of research integration should be achieved in this field and that the existing research fragmentation characteristics should be removed [72]. Climate resilience is a crucial theme of community resilience construction, and research methods mostly use assessment frameworks and indicators as the starting point to explore specific resilience-improvement strategies. DasGupta et al. constructed a five-dimensional framework for assessing the climate-related resilience of coastal administrative blocks of Indian Sundarbans and emphasized the vital function of institutional intervention in effectively building climate resilience in coastal areas [73]. Climate resilience-building is an important aspect of current community development and a major capability that needs to be strengthened urgently within the international community and cities. UN-Habitat has developed a “planning for climate change toolkit” for urban communities in low- and middle-income countries to better understand, assess and respond to climate change at the local level [74]. The Asia-Pacific network for global change research has developed a “community resilience tool” for rural communities [75]. The Intergovernmental Panel on Climate Change (IPCC), established by the United Nations Environment Programme (UNEP) and the World Meteorological Organization (WMO) (Washington, USA), also provides scientific guidance and countermeasures for global climate change risks. The impact of climate change is a key concern at different levels of society. Enhancing climate resilience requires collaboration among different subjects.

Topic three: Social capital mechanism (cluster two).

Social capital is one of the most relevant topics in the process of community resilience development. As the largest cluster in the intellectual base, it is also the second-largest cluster among the research hotspots, fully demonstrating that the role of the social capital mechanism is an important aspect of academia’s focus on improving community resilience. Social capital refers to the ability and willingness of community members to participate in actions aimed at community goals, as well as the process of participation, that is, individuals acting individually or collectively in community organizations, groups, and networks [76]. Aldrich regarded social capital as the “core engine of recovery” for the community in disasters and believed that survivors who have connections with powerful social networks can obtain necessary information and support and recover faster than those without social network connections [43]. Previous scholars divided social capital into three types, including bonding social capital (associations among similar members of a group or community); bridging capital (associations among dissimilar members); and linking capital (connections with other members, institutions, or networks that have greater power or authority) [77]. For individuals affected by disasters, bonding social capital is the most common form of a social network. It is practical to receive assistance from family and friends when disasters come. For example, Chinese families with larger Spring Festival networks were more likely to rebuild their homes in 2008 [78]. Pfefferbaum, B also called for enhancing community disaster resilience by strengthening social capital and discussed the significance of social capital generated by building team relationships and improving social networks and social connections in enhancing resilience [79]. Because of the characteristics of social capital, most previous studies have studied the role of social capital in a certain disaster using questionnaires or interviews. In recent years, studies on social capital have focused more on measuring and evaluating social capital through publicly available data. Kyne and Aldrich used publicly available indicators to obtain the social capital index (SoCI), which was applied to counties in the home states of the United States to measure three types of social capital, providing a specific method for the measurement of social capital [80]. As the central mechanism of community resilience research, the empirical research method of social capital gradually shows important value [78,81]. Specifically, studies on the mechanism of social capital increasingly rely on publicly available data rather than questionnaires or interviews.

Topic four: Macro environment and community disaster reduction policies (cluster three and cluster five).

The continuous advancement of globalization accelerated the opening of community boundaries in geographical, socio-cultural, political, and economic fields [82]. At the community level, these disruptions from globalization pose a huge challenge to dealing with environmental and social change. In the macro environment, some scholars began to pay attention to the impact of the globalization process on community resilience. Wilson et al. believed that capital and economic globalization are important reasons for the blurring of community boundaries [82]. Community resilience tends to be negatively affected by globalization processes, with community members pursuing vertical integration (global economy) rather than horizontal integration (economic interconnections within and between communities). The key to maximizing resilience is to strike the right “balance” between communities and globalization [82]. Thus, how national policies guide communities to build resilience has become a major concern for disaster managers and politicians. Geoff utilized the policy corridor theory to analyze the possible impact of national policies on community resilience and explained that building strong community resilience is often an endogenous process and strengthening bonding and bridging social capital is beneficial. However, community-level actors cannot always play a role in resilience-building alone, and a combination of national and grass-roots approaches is the best way to strengthen resilience [83]. To explore the effectiveness of government mitigation policies on community resilience, Ji and Lee compared disaster loss data in counties receiving the Hazard Mitigation Grant Program (HMGP) in the United States. The results showed that counties that participated in HMGP were less likely to suffer property damage in future disasters [84]. 

Topic five: Community resilience evaluation index system (cluster six).

Facing sudden disaster risks, the integrated resilience assessment system has become an important measure to identify the vulnerability factors of the community and improve the adaptability and resilience level of the community. Resilience assessment is the latest development in community resilience. Especially in the last 10 years, there has been a great deal of related research. By reviewing the evaluation criteria of previous literature, community resilience assessment generally includes five dimensions: environment, society, economy, built environment and infrastructure, and system. Community resilience is usually assessed by quantitative methods supported by data or qualitative methods based on public perception and judgment by experts and scholars. Commonly used assessment tools mainly include scorecards, indicators, models, and toolkits. Indicators are more commonly used in research [17]. Orencio et al. used Delphi technology to invite 20 local decision-makers to explore the vulnerability standards and related factors affecting coastal communities through the AHP method [85]. The results show that the impact of environmental and natural resource management, sustainable livelihood, social protection, and planning regimes is the most significant, and the obtained comprehensive index has reference value for the resilience-building of local communities [85]. Based on the development of the Disaster Resilience of place model [14], Cutter, S.L. constructed the index of community resilience baseline characteristics [15] and formed the final community resilience baseline index (BRIC) [86]. The toolkit contains designated scorecards, indicators, and models to measure the mechanism and process of resilience. It is a community resilience assessment method that should be promoted in the future. Schoch-Spana et al. developed the COPEWELL Rubric, a participatory, bottom-up self-assessment tool for community resilience developed in collaboration with community users and national thought leaders to predict the post-disaster operations and resilience of different communities through the joint efforts of different participants [87]. Pfefferbaum et al. developed a Toolkit for Community Resilience (CRAT), including the CART assessment survey, key informant interviews, data collection framework, and community dialogue conversations, neighborhood infrastructure maps, community ecological maps, and SWOT analysis [88]. It is a process of community empowerment through information, communication, and assistance to identify problems, solve problems, and plan activities [88]. It can be concluded that both indicators and other community resilience assessment tools play an important role in improving community resilience. In the future, systematic resilience assessment will become more popular in combining different tools according to the needs of different dimensions.

## 5. Conclusions

Based on the obtained literature data on community resilience over the past two decades, this study utilized bibliometrics and visualization methods to present the characteristics of the knowledge distribution of community resilience research from both the macro and micro levels. Meanwhile, the intellectual bases and research hotspots of community resilience research were explored. The following conclusions are drawn:

1. Community resilience research has gone through the initial stage, slow development stage, and rapid growth stage in terms of publications. After 2010, literature on community resilience began to increase gradually. Especially after 2013, the number of published papers has achieved rapid growth. In 2020, the rate reached 475 papers/year.
2. From the perspective of macro-geographical distribution, the United States, Australia, and the United Kingdom have made the most contributions to community resilience research. The global geographic heat map shows that North America, Oceania, Europe, and Asia are hot spots for community resilience research, while most South American and African countries still need to strengthen their output. In terms of institutional distribution, Colorado State University and Texas A&M University are the most influential institutions in community resilience research.
3. From the perspective of the micro-knowledge distribution characteristics, the categories of ecological environment, water resources, and geography are the subject areas with the largest proportions in interdisciplinary resilience research. Global Environmental Change—Human and Policy Dimensions is the most widely researched journal in community resilience research. The most prolific author is John W. van de Lindt. Although some core authors do not publish the most articles, their theories or research have significantly impacted the development of community resilience, such as Cutter S.L. and Norris F.H.
4. Through literature co-citation analysis and high-frequency keyword clustering analysis, this study revealed the intellectual bases and research hotspots in community resilience research. Community resilience research is mainly divided into the following seven sections: social capital mechanism, evolution of resilience knowledge, earthquake resistance and disaster mitigation, substance abuse group research, rural community resilience development, resilience-building in the least-developed countries, and emergency preparedness. The seven sections form the intellectual basis of community resilience research. The cluster analysis of high-frequency keywords obtains nine clusters. Through further merger and integration, five research hotspots in the community resilience field were revealed, including the concept of resilience, climate resilience, social capital mechanism, macro-environment, and community disaster-reduction policies, and an evaluation index system of community resilience.
5. Focusing on future development, community resilience research will continue to conduct more in-depth research in hotspots and fronts fields. First, in terms of resilience assessment, more raw data and publicly available secondary data will be explored to consummate the concept and corresponding mechanism, and the needs and priorities of different communities will be focused on to improve the community resilience assessment system. Thus, comprehensive resilience assessment frameworks and methods will be further integrated. Second, social capital continues to be the central mechanism of community resilience research, and its significance and measures will be further expanded. Third, the research on the intervention factors of the psychological resilience of special groups and their behaviors may be transferred to new groups. Fourth, global projects will sustain the use of community interventions to advance community resilience practices and enhance the resilience of communities to combat major risks. Fifth, with the emergence of new natural disasters and man-made disasters, communities will play a vital role in coping with total disasters in the future. Therefore, exploring the construction of the community resilience field in total disasters is also one of the possible directions for breakthroughs in the future.
6. However, there are certainly several limitations to this study that need to be acknowledged. Bibliometric analysis can bring about some interesting clustering results in terms of authors, locations, and themes. Intellectual bases and hotspots of community resilience were also analyzed based on the bibliometric analysis in Section 4. Unfortunately, bibliometric studies are still not as in-depth as traditional literature reviews. Furthermore, the keywords may be partially useful in the bibliometric analysis because there are some common phraseologies around the general theme of resilience. Therefore, the author’s understanding of the scientific field should be added based on bibliometrics.

## Figures and Tables

**Table 1:** Top 10 countries/regions according to publications. Note that TC and TC/P represent the total citations and the average of citations per paper for a country, respectively.

Rank	Country	Publications	Percentage	Centrality	TC	TC/P	Starting Year
1	USA	875	28.65%	0.26	21,168	24.19	2001
2	Australia	287	9.40%	0.32	4733	16.49	2003
3	England	207	6.78%	0.94	4351	21.02	2002
4	Canada	150	4.91%	0.07	3430	22.87	2003
5	China	135	4.42%	0.08	1331	9.86	2004
6	New Zealand	101	3.31%	0	1920	19.01	2001
7	Italy	95	3.11%	0.07	1284	13.52	2011
8	The Netherlands	78	2.55%	0	692	8.87	2010
9	Japan	74	2.42%	0	857	11.58	2009
10	Germany	59	1.93%	0.44	934	15.83	2004

**Table 2:** Top 10 productive institutions according to publications.

Rank	Institutions	Publications	Centrality	Starting Year	TC	TC/P
1	Colorado State University	51	0.08	2008	745	14.61
2	Texas A&M University	38	0.22	2008	770	20.26
3	The University of Queensland	37	0.03	2007	1134	30.65
4	Louisiana State University	28	0.02	2008	920	32.86
5	Oregon State University	26	0	2009	253	9.73
6	The University of Melbourne	25	0.08	2009	310	12.40
7	University of Washington	25	0.23	2003	1689	67.56
8	University of California Los Angeles	24	0.11	2009	443	18.46
9	RAND Corp	23	0.02	2009	373	16.22
10	Oklahoma State University	23	0.09	2015	368	16.00

**Table 3:** Top 10 categories in community resilience research from 2001 to 2020.

Rank	Category	Publications	Centrality	Starting Year	TC	TC/P
1	Environmental Sciences and Ecology	691	0.16	2001	13,792	19.96
2	Water Resource	328	0.04	2001	5149	15.70
3	Geosciences, Multidisciplinary	302	0	2001	4965	16.44
4	Meteorology and Atmospheric Sciences	293	0	2001	4842	16.53
5	Science and Technology—Other topics	290	0.45	2002	5655	19.50
6	Public, Environmental and Occupational Health	263	0.32	2001	3434	13.06
7	Engineering	193	0.18	2003	3973	20.59
8	Geography	150	0	2004	527	34.85
9	Public Administration	141	0.06	2002	3817	27.07
10	Business & Economics	136	0.26	2008	2024	14.88

**Table 4:** Top 10 highly cited journals in community resilience research from 2001 to 2020.

Rank	Journal	Citations	Total Link Strength	Host Country/Region
1	Global Environmental Change-Human and Policy Dimensions	2257	82,562	England
2	Natural Hazards	1774	56,064	USA
3	Ecology and Society	1744	79,466	Canada
4	International Journal of Disaster Risk Reduction	1092	32,633	The Netherlands
5	Disasters	1063	32,229	England
6	Science	989	42,654	USA
7	American Journal of Community Psychology	944	24,572	USA
8	Society Natural Resources	861	29,194	USA
9	Proceedings of the National Academy of Sciences of the United States of America	777	36,047	USA
10	Nature	646	26,246	England

**Table 5:** Top 10 productive authors in community resilience research from 2001 to 2020. Note that the starting year represents the year when the article was first published.

Rank	Author	Publications	Starting Year	Institution	Country	H-Index
1	John W van de Lindt	20	2016	Colorado State University	USA	28
2	Shaul Kimhi	14	2016	Tel Hai Academy College	ISRAEL	15
3	Yohanan Eshel	14	2016	Tel Hai Academy College	ISRAEL	11
4	Hussam Mahmoud	14	2018	Colorado State University	USA	13
5	Naiyu Wang	11	2016	Zhejiang University	CHINA	19
6	Anita Chandra	10	2012	Cambridge Inst Therapeut Immunol & Infect Dis CIT	ENGLAND	22
7	Peihui Lin	10	2016	Zhejiang University	CHINA	8
8	Suzanne Wilkinson	9	2016	Massey University	NEW ZEALAND	17
9	Bruce R Ellingwood	8	2016	Colorado State University	USA	49
10	Mooli Lahad	8	2013	Tel Hai Academy College	ISRAEL	10

**Table 6:** Top 10 highly cited authors in community resilience research from 2001 to 2020.

Rank	Cited Author	Frequency	Centrality	Starting Year	Institution	H-Index
1	Cutter SL	658	0.06	2008	University of South Carolina System	41
2	Norris FH	640	0.06	2008	Dartmouth College	64
3	Adger WN	507	0.25	2002	University of Exeter	60
4	Folke C	381	0.11	2008	Stockholm University	94
5	Berkes F	375	0.08	2006	University of Manitoba	46
6	Holling CS	350	0.11	2008	University of Florida	37
7	Paton D	262	0.14	2007	University of Canberra	32
8	Walker B	254	0.1	2008	Australian National University	58
9	Bruneau M	215	0.07	2010	State University of New York (SUNY) System	33
10	Aldrich DP	212	0.01	2012	Northeastern University	14

**Table 7:** Top 10 highly cited documents in community resilience study from 2001 to 2020.

Rank	Title	Cited Frequency	Author	Centrality	Document Type	Year
1	Community Resilience as a Metaphor, Theory, Set of Capacities, and Strategy for Disaster Readiness	244	Norris, FH; Stevens, SP; Pfefferbaum, B; Wyche, KF; Pfefferbaum, RL	0.05	Review	2008
2	Community Resilience: Toward an Integrated Approach	217	Berkes, F; Ross, H	0.02	Article	2013
3	A place-based model for understanding community resilience to natural disasters	164	Cutter, SL; Barnes, L; Berry, M; Burton, C; Evans, E; Tate, E; Webb, J	0	Article	2008
4	Community Resilience: An Indicator of Social Sustainability	127	Magis K	0.31	Article	2010
5	Building Resilience: Social capital in post-disaster recovery	125	Aldrich DP	0.04	Book	2012
6	The geographies of community disaster resilience	121	Cutter, SL; Ash, KD; Emrich, CT	0.21	Article	2014
7	Social Capital and Community Resilience	108	Aldrich, DP; Meyer, MA	0	Article	2015
8	Disaster Resilience Indicators for Benchmarking Baseline Conditions	108	Cutter, SL; Burton, CG; Emrich, CT	0.46	Article	2010
9	Resilience and disaster risk reduction: an etymological journey	80	Alexander, DE	0.02	Article	2013
10	Measuring Capacities for Community Resilience	79	Sherrieb, K; Norris, FH; Galea, S	0.28	Article	2010

**Table 8:** Top 30 keywords with frequency and centrality.

Rank	Keywords	Frequency	Centrality	Rank	Keywords	Frequency	Centrality
1	Community resilience	976	0.16	16	Sustainability	108	0.11
2	Resilience	658	0.15	17	Hazard	99	0.01
3	Vulnerability	338	0.13	18	Disaster resilience	97	0.17
4	Climate change	326	0.12	19	Preparedness	96	0.06
5	Disaster	313	0.02	20	Earthquake	95	0.04
6	Framework	247	0.04	21	Governance	90	0
7	Adaptation	239	0.14	22	Capacity	90	0
8	Risk	219	0.06	23	Perception	89	0
9	Management	210	0.12	24	Adaptive capacity	82	0.1
10	Community	151	0.06	25	Perspective	80	0.07
11	Recovery	150	0.02	26	Indicator	80	0.18
12	Model	150	0	27	Social vulnerability	79	0
13	System	142	0.06	28	Natural disaster	76	0
14	Impact	136	0.2	29	Policy	75	0
15	Health	119	0.21	30	Strategy	66	0.14

## Data Availability

The manuscript has no data associated with it other than that presented in Table 1, Table 2, Table 3, Table 4, Table 5, Table 6, Table 7 and Table 8.
